# Parameterization for *In-Silico* Modeling of Ion Channel Interactions with Drugs

**DOI:** 10.1371/journal.pone.0150761

**Published:** 2016-03-10

**Authors:** Jonathan D. Moreno, Timothy J. Lewis, Colleen E. Clancy

**Affiliations:** 1 Division of Cardiology, Department of Medicine, Barnes-Jewish Hospital, Washington University in St. Louis, St. Louis, MO, United States of America; 2 Department of Mathematics, University of California Davis, Davis, CA, United States of America; 3 Department of Pharmacology, University of California Davis, Davis, CA, United States of America; Georgia State University, UNITED STATES

## Abstract

Since the first Hodgkin and Huxley ion channel model was described in the 1950s, there has been an explosion in mathematical models to describe ion channel function. As experimental data has become richer, models have concomitantly been improved to better represent ion channel kinetic processes, although these improvements have generally resulted in more model complexity and an increase in the number of parameters necessary to populate the models. Models have also been developed to explicitly model drug interactions with ion channels. Recent models of drug-channel interactions account for the discrete kinetics of drug interaction with distinct ion channel state conformations, as it has become clear that such interactions underlie complex emergent kinetics such as use-dependent block. Here, we describe an approach for developing a model for ion channel drug interactions. The method describes the process of extracting rate constants from experimental electrophysiological function data to use as initial conditions for the model parameters. We then describe implementation of a parameter optimization method to refine the model rate constants describing ion channel drug kinetics. The algorithm takes advantage of readily available parallel computing tools to speed up the optimization. Finally, we describe some potential applications of the platform including the potential for gaining fundamental mechanistic insights into ion channel function and applications to *in silico* drug screening and development.

## Introduction

As our understanding of ion channel biophysics has increased, so too have mathematical models been continually improved to better recapitulate experimentally observed kinetics. Ion channel models have been used extensively to explain and predict normal channel behavior, but also have been very useful to provide insights into aberrant function resulting from mutations in genes encoding ion channels, as well as to predict drug interactions with ion channels.

This paper is meant as a tutorial for parameter optimization of ion channels that highlights benefits and common pitfalls associated with computational modeling. We describe all the necessary steps in a process that includes experimental data extraction, implementation of an automatic parameter optimization routine, and the use of parallel computing tools and a multi-core chipsets found in most desktop computers. We present as an example, an overview of the cardiac Na^+^ channel and the development of a Markov-chain model, which allows for explicit representation of conformational states and rates of transition between them. Using the model, we then present an example application of the tool: development of a drug-channel interaction model used for prediction of emergent pharmacology and safety testing.

### Determination of channel model structure

The human cardiac voltage-gated Na^+^ channel is a macromolecular complex that assumes distinct conformational changes in response to voltage perturbations (as occur during the action potential) [1]. Early models of the Na^+^ channel consisted of 3 states: resting (closed), activated (open), and inactivated [2]. As understanding of cardiac Na^+^ channel structure and function has been refined through increasingly available experimental data, models describing the cardiac Na^+^ channel [3–7] [8] have become more complex. We recently developed a model for the cardiac Na^+^ channel Na_V_1.5 that includes 8 distinct states [3] to capture the complex features of Na^+^ channel kinetics, including time- and voltage-dependent activation [9], inactivation (both open and closed state) [10], a multi-exponential recovery from inactivation (fast and slow) [10], and channel mean open time [11].

In response to a voltage depolarization, the cardiac Na^+^ channel transits from a nonconducting closed state to a conducting open state. Soon after opening, the channel quickly inactivates through a conformation change to a nonconducting absorbing state that is only relieved by repolarization. Repolarization also promotes channel transit back to a resting closed state [1,12]. The transitions between these states are voltage dependent [13]. The model we previously described [3] contains 11 independent rate constants (18 parameters), and one rate (β2) constrained by microscopic reversibility, a thermodynamic constraint that at equilibrium, for any closed loop in the system, the product of the forward rate constants must equal the product of the reverse rate constants [14].

**Fig 1:**
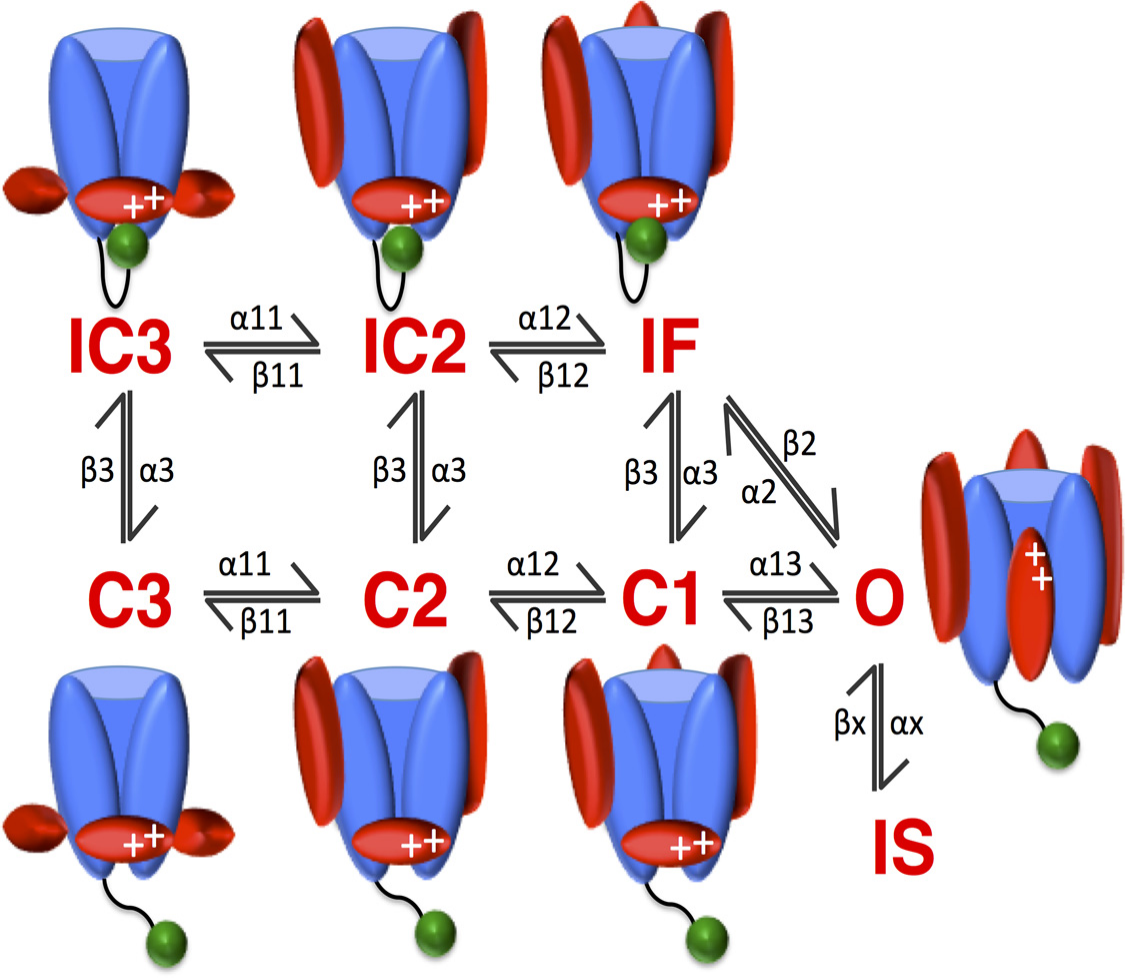
Schematic of the Wild-Type Na^+^ Channel. The wild-type Na^+^ channel formulation contains 8 states: 3 closed states (C3, C2, C1), 1 open state (O), a fast- and slow-inactivated state (IF and IS, respectively), and two close-inactivated states (IC3, IC2). Included are cartoon representations of the gating structure closely associated with the kinetic state of the channel. Note the movement of the S4 voltage sensors (red ovals around the channel pore) as the channel traverses the closed states (C3 → C2 → C1 → O). Note also the fast inactivation gate (green ball), the III-IV linker, occluding the channel pore on movement from O → IF and C → IC, IF [17–19].

The model structure in **Fig 1** (and the corresponding rate constants in **Table 1**) is a state-specific model that includes coupling between channel conformational states through voltage dependent rate constants. This form differs from the Hodgkin-Huxley type model, which is based on the assumption of independence between gates (i.e. activation, inactivation). The coupled state-based model is an attempt to explicitly represent specific, experimentally determined channel conformations and the movement between them [15]. This may be especially useful for predicting behaviors that are state dependent, such as the effects of mutations and how ion channels interact with drugs and toxins [16].

**Table 1:** Parameters to be optimized in the drug free Na+ channel.

IC3 →IC2, C3→C2	α11 = 1/(a11_v1*exp(-V/***a11_v2***))
IC2→IF, C2→C1	α12 = a12*α11
C1→O	α13 = a13*α11
IC2→IC3, C2→C3	β11 = 1/(b11_v1*exp(V/***b11_v2***))
IF→IC2, C1→C2	β12 = b12*β11
O→C1	β13 = b13*β11
IC3→C3, IC2→C2, IF→C1	α3 = a3_v1*exp(-V/***a3_v2***)
C3→IC3, C2→IC2, C1→IF	β3 = b3_v1*exp(V/***b3_v2***)
IF→O	β2 = (α13* α2* α3)/(β13* β3)
O→IF	α2 = a2_v1*exp(V/***a2_v2***)
O→IS	αx = ax*α2
IS→O	βx = bx*α3

**Fig 2:**
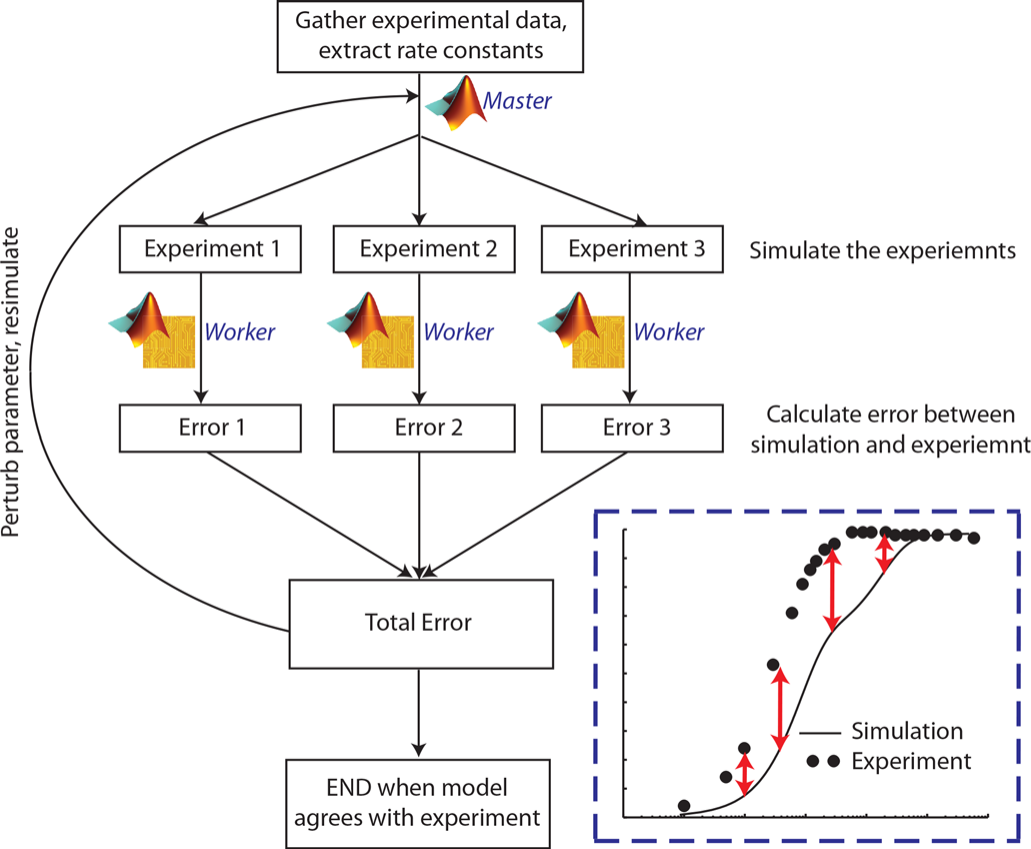
Schematic of the optimization procedure. The first step in the algorithm gathers the experimental data; experiments are then simulated. The algorithm compares the difference, or error, between experiment and what the model predicts (see inset: red arrows indicate error, the simulated experiment is denoted by the solid line, the experiment data are the dots). The total error is then summed. The routine perturbs the parameter set, and iterates again. This routine finishes when the model agrees sufficiently well (defined as the change in cost function between iteration *n* and *n+1*) with the experiment. In other words, when the error between what the model predicts and the actual experiment falls below a predefined value.

*Note, these parameters correspond to the labeled transition rates in **Figs 1 and 2**. The rates are in (ms^-1^). As mentioned in the text, there are 11 independent rate constants, with one constrained by microscopic reversibility (β2). Within the 11 independent rate constants, there are 16 free parameters to be optimized (shown in ***italicized bold***). A note on naming: “a11_v2” is a11 variable 1, and “a11_v2” is a11 variable 2.

### Brief overview of optimization methods

One of the major challenges of complex computational models is the large number of free parameters that must be determined [20]. How best to fit these parameters to experimental data has been the subject of many studies [21–25]. There have been multiple parameter optimization methods used for ion channel models including principal-axis fitting [26], maximum-likelihood estimation [27,28], genetic algorithms [21,29,30], and the widely used empirical “hand-tuning” of free parameters [6,21,31–33], whereby parameters were tweaked incrementally to achieve a qualitatively acceptable fit to the data, as defined by the investigator. The empirical adjustment method relies primarily on operator intuition, developed through analysis and interpretation of many data sets. It is subjective, functionally slow, and does not satisfy the increasingly stringent requirements for robustness and reproducibility.

In contrast to hand fitting, automatic parameter optimization procedures can survey a much larger parameter space, determine quantitatively best fits to multiple voltage-clamp datasets simultaneously, are reproducible, are “operator intuition” independent, relatively easy to implement, and if given the right constraints on the optimization criteria (e.g. positive rate constants, topologically “allowed” transitions based on an understanding of channel structure and gating etc.), can achieve a physiologically relevant parameter set. The disadvantage of automated optimization is that the automatically derived parameter set may not necessarily reflect known properties of channel gating [21]. For both automatic and “manual” optimization, it is not always possible to definitively identify a unique global minimum; multiple local minima may be identified that correspond to a parameter set that “fits” the data with sufficiently meaningful precision (within the standard error of measurements). With appropriate validation, a sufficiently detailed representation of channel gating that represents a local minimum may be all that is required. As will be discussed, there are tradeoffs between efficiency, parameter identifiability, ease of implementation, and results that are sufficient to answer the scientific question. For a detailed review on parameter identifiability and necessary conditions for unique parameter sets, see Fink and Noble [22] and Dokos and Lovell [23].

We have implemented a direct search simplex algorithm developed by Nelder and Mead [34] that attempts to minimize a scalar-valued cost function of *n* real variables using only function values, without any derivative information [35]. Also called the “amoeba” method, the algorithm crawls through parameter space creating ever-smaller simplexes until a local minimum is found. We chose the Nelder Mead method as it is among the easiest to implement, relatively robust and does not require derivative information of the cost function. The goal for the method was accessibility, so that students, trainees and non-experts can apply the method to models of interest.

Our implementation takes advantage of a multi-core chip architecture found in many desktops and the Parallel Computing toolbox™ within MATLAB to speed up computation time. The Parallel toolbox is an add-on package for MATLAB that is particularly suited to this type of optimization problem, given the many independent tasks that can be run simultaneously. In this fork-join method, the model code sends each “experiment” (a voltage clamp electrophysiology protocol in a cell expression system) to be simulated to a separate “*worker*” (or MATLAB computational engine running independently of the desktop MATLAB session) which can be computed simultaneously and independent from the other experiments (a ‘fork’); the results are then combined into a larger objective function (a ‘join’). One distinct advantage is that the toolbox does not require CUDA™ or MPI programming knowledge, and is easy to implement in pre-existing code.

## Methods

### Extraction of rate constants from the experimental literature

We begin by describing as an example a method of rate constant extraction for the cardiac Na^+^ channel model [3]. Experimental function data suggest that the Na^+^ channel transits through a series of closed states upon activation to an open conducting state [1,12]. To assign each of the kinetic transitions listed in **Table 1** a rate, experimental function data designed to isolate specific transitions are gathered from expressed Na^+^ channels in single cells.

The rates for activation (**α11, α12, α13** corresponding to the states C3 →C2→C1→O), and deactivation (**β13, β12, β11** corresponding to O→C1→C2→C3 –see **Fig 1**) were based on a single exponential for the main activation parameter, α_11_, and main deactivation parameter β_11_. The remaining two activation and deactivation rates were linear combinations of α_11_ and β_11_. Thus, the activation and deactivation rates were of the following form:

$$\begin{array}{l} \alpha_{11}(V)=\frac{1}{a11\_v1\;*\;\exp(-V/a11\_v2)}\\ \alpha_{12}(V)=a12\;*\;\alpha_{11}\\ \alpha_{13}(V)=a13\;*\;\alpha_{11}\\ \beta_{11}(V)=\frac{1}{b11\_v1\;*\;\exp(V/b11\_v2)}\\ \beta_{12}(V)=b12\;*\;\beta_{11}\\ \beta_{13}(V)=b13\;*\;\beta_{11} \end{array}$$

The initial guesses for parameters of rates α11 and β11 (***a11_v1*, *a11_v2*, *b11_v1*, *b11_v2***) were taken from Mitsuiye and Noma [36]; the remaining parameters (***a12*, *a13*, *b12*, *b13***) were initially set to 1 (thus equal to α11, β11 respectively). A note on nomenclature: the (_v1, _v2) in the above variables (e.g. a11_v1, a11_v2), stand for ‘variable 1’, ‘variable 2’). The rates that are voltage dependent are denoted in the equations with “*V*” (capital V). Thus, α_11_(V) has two independent variables to be optimized (a11_v1, a11_v2).

The recovery from inactivation rate constant (**α3** corresponding to the states IC3, IC2, IC1 → C3, C2, C1 –see **Fig 1**) was from fitting recovery from inactivation at 3 different voltages (-80mV, -100mV, -120mV) with a double exponential function of the form:

$$y=C1-A1\;*\;\exp(-t/\tau_{1})-A2\;*\;\exp(-t/\tau_{2})$$

The rate constant α_3_ is proportional to 1/τ_1_. Using the methods of Colquohn and Hawkes [37] to reduce the number of free parameters, a voltage dependent rate constant of the form below was derived with two parameters for optimization (**a, b**) (see **S1 Supplementary Information**):

$$\alpha_{3}(V)=a\;*\;\exp(-V/b)$$

Closed state inactivation (**β3** corresponding to C3, C2, C1 → IC3, IC2, IC1 –see **Fig 1**) was from Goldman [38], and included two parameters for optimization (**a, b**) and was of the form:.

$$\beta_{3}(V)=a\;*\;\exp(V/b)$$

Inactivation from the open state (**α2** corresponding to O → IF–see **Fig 1**) was found by fitting time constants of ensemble averaged current decay data from Yue and Marban [13] to an exponential equation with two parameters for optimization (**a, b**) of the form:

$$\alpha_{2}(V)=a\;*\;\exp(V/b)$$

Recovery from fast inactivation back to the open state (**β2** corresponding to IF → O–see **Fig 1**) was constrained by microscopic reversibility (as detailed above) and was of the form:

$$\beta_{2}=\frac{\alpha_{13}\;*\;\alpha_{2}\;*\;\alpha_{3}}{\beta_{13}\;*\;\beta_{3}}$$

Slow inactivation from the open state (**αx** corresponding to O → IS–see **Fig 1**) was from Lawrence et al. [39] which measured slow inactivation at -20 mV = 0.111/ms, corresponding to ≈ α2/20. Recovery from slow inactivation (**βx** corresponding to IS → O–see **Fig 1**) was initially set at a3/45 (empirically). See **S1 Supplementary Information** for the derived initial conditions, and the optimized parameters.

### Overview of the optimization routine

Electrophysiological experiments that were used in the optimization procedure were chosen to cover a wide range of channel gating kinetics over the range of physiologically relevant voltages. At least one experimental protocol that attempted to isolate each transition in the model was included in the optimization. For example, optimization of the wild-type Na^+^ channel model includes experimental protocols that include steady-state availability, steady state activation, recovery from 1-pulse inactivation, recovery from multiple pulse inactivation, time constant of current decay, and channel mean open time. Fink and Noble [22] suggest a unified protocol to represent “the normal range of voltage-clamp steps used to characterize ion channel dynamics”. Although this would be ideal, most often models are built based on datasets that derive from previously published works from different laboratories under different conditions and measured by different “hands”. Our strategy derives from this reality and should allow the modeler to build a modular framework that includes multiple separate experiments. In some instances, it might be preferable to utilize data from varied sources so as not to build a model of a particular data set, but rather something more representative of general behaviors captured by many experimentalists. This should help to minimize any systematic errors in measurement as well. The model may even, in some instances, be useful to reconcile or explain disparate data sets.

Individual objective functions from each *in silico* experiment are defined as the sum of squared errors between experiment and simulation, and normalized to the sampling rate of each protocol. For example, steady state inactivation simulates channel availability at 9 different voltages (9 data points), whereas mean open time simulates just 1 point. Normalizing to this sampling rate ensures equal weighting between protocols such that no kinetic transition in the model is disproportionately favored. Notably, sequential optimization constrains the model to a particular parameter space, thereby implicitly weighting the first protocol optimized disproportionately more than the later protocols. Thus, equal weighting is further accomplished by optimizing each protocol in parallel rather than sequentially. Finally, individual, normalized objective functions are summed to an overall objective function, which is ultimately passed to the minimization routine.

The minimization routine requires specific inputs including (1) the optimization algorithm (in our case the Nelder Mead algorithm); (2) a vector of initial guesses of the extracted rate constants (see **S1 Supplementary Information** for initial rates vector); (3) a vector of specific options for the algorithm to be used (e.g. tolerance, maximum number of function iterations etc.); and (4) the overall objective function to be minimized. For simplicity, we chose a bounded Nelder Mead optimization algorithm that only permits positive rate constants, and does not require any derivative information. However, other optimization algorithms can be substituted (e.g. Quasi-Newton, Interior-Point, Trust Region Reflective, Genetic Algorithm, Simulated Annealing, among others). See **Fig 2** for a general schematic of the optimization routine, and **Table 2** for example code.

**Table 2:** Example Code and Key Commands.

parpool('local',5);	*Open a parallel pool of 5 MATLAB workers (independen*
	*t MATLAB engines to send each experiment to)*
mex main_SSA.cpp	*Compile C++ files within MATLAB*. *Each file is*
mex main_ACT.cpp	*a separate experiment to be simulated (e*.*g*. *SSA–stead*
	*y state availability*, *ACT–activation)*. *If possible*, *the number of workers in ‘parpool’ should equal the number of experiments to be simulated*.
Inputs = [	*Provide a vector of initial guesses*.
0.1027;	
…	
9.3;	
0.250];	
LB = [0; 0;…; 0];	*Provide bounds on the parameter values (e*.*g*. *strictl*
UB = [Inf; Inf;…; Inf];	*y positive values*: *LB–lower bound*, *UB–upper bound)*
options = optimset	*Provide a list of options to the optimization algorithm*
('TolFun', 1e-2, 'MaxIter', 2);	*(e*.*g*. *Maximum iterations*, *tolerance on the function etc*.*)*
Inputs_Final = fminsearchbnd (@WT_CHANNEL, Inputs,lb,ub, options);	*Main function call to the minimization algorithm*. *Use the ‘fminsearchbnd’ (Bounded Nelder-Mead) algorithm to compute the minimum of the function ‘WT_CHANNEL’*, *with initial guesses provided by the ‘Inputs’ vector*, *bounded by ‘LB’*, *and ‘UB’*, *using the ‘options’ provided*.
parfor i = 1:n;	*Create a parallel ‘for’ loop (‘parfor’) to send each*
if i = = 1	*experimental protocol to a separate MATLAB worker*.
main_SSA(Inputs);	*This step simulates each protocol with the vector of*
end	*‘Inputs’*
if i = = 2	
main_ACT(Inputs);	
end	
…	
end;	

## Results

### Simulation of Na^+^ channel kinetics: pre- and post-optimization

To capture multiple aspects of physiologic cardiac Na^+^ channel gating, our optimization routine included 6 common protocols that capture the diverse voltage- and time-dependent properties of the channel: steady state availability (SSA), steady state activation (ACT), recovery from inactivation at -100 mV (RFI), recovery from use-dependent block (RUDB), time to 50% decay of Na^+^ current (Tau50%), and mean open time at -30 mV (MOT).

**Fig 3:**
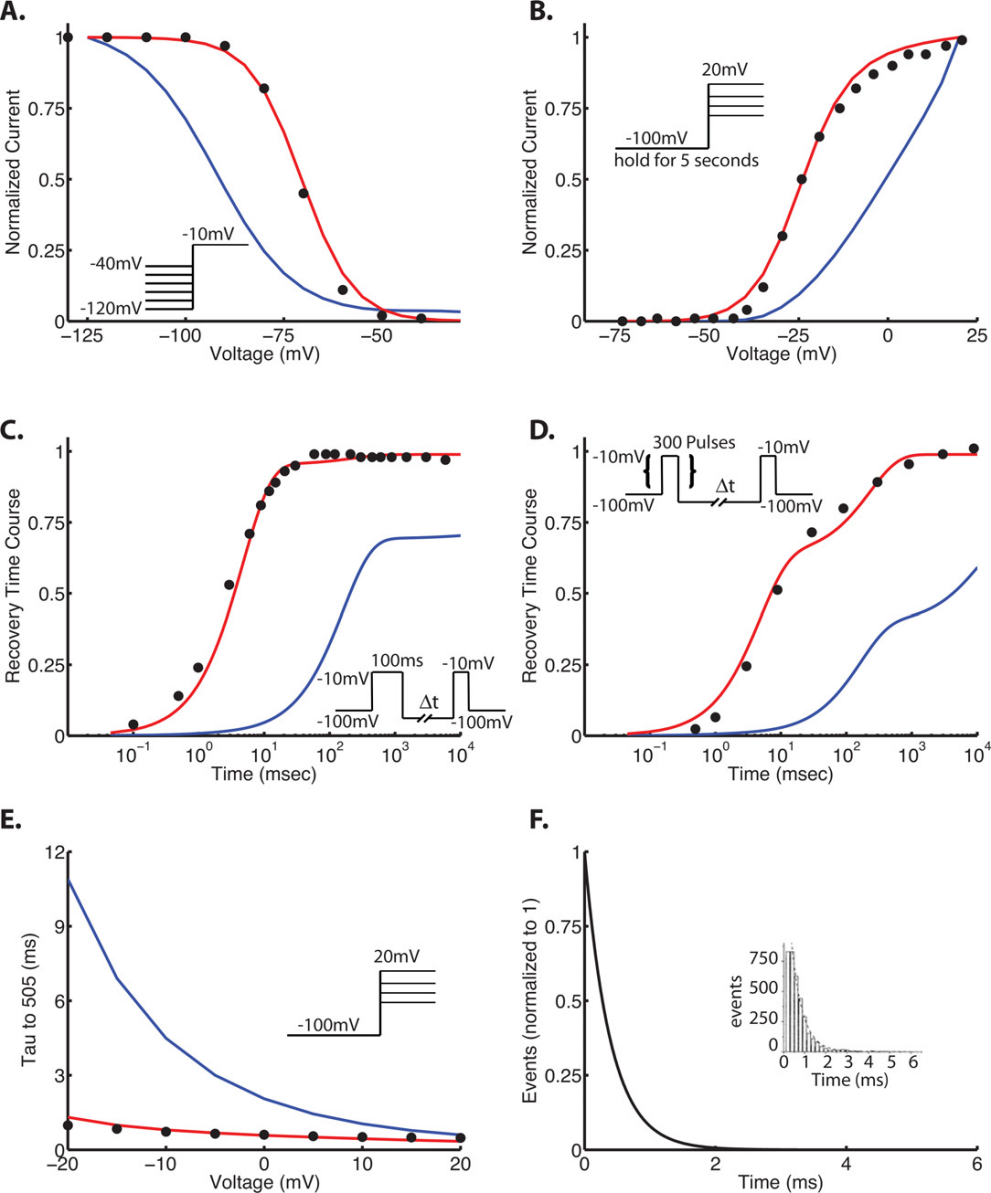
Wild-type Na^+^ channel kinetics–pre- and postoptimization. In each panel, the points are experiment, and the lines are simulation. Blue traces indicate preoptimization using initial guesses as described in the text; red traces indicate the optimized parameters. The protocols are as follows: steady state availability (or inactivation) (Panel A), steady state activation (Panel B), recovery from inactivation at -100 mV (Panel C), recovery from use-dependent block (Panel D), and time to 50% decay of Na^+^ current (Tau50%) (Panel E). The model was further constrained by mean open time at -30 mV. Data are from [3,9,10]. Voltage protocols are shown as insets.

Extraction of rate constants from the literature (described above) yielded initial fits to 6 protocols as shown in **Fig 3** (blue traces). These initial fits are not surprising, as the rate constants were extracted from different laboratories with different experimental conditions and protocols, and ultimately serves to show the biological variability and noise from preparation to preparation [23]. It also clear from the initial fits to the data that individual protocols do not necessarily isolate one specific kinetic transition, but rather capture simultaneous gating processes [22].

### Optimized drug free Na^+^ channel model

**Fig 4:**
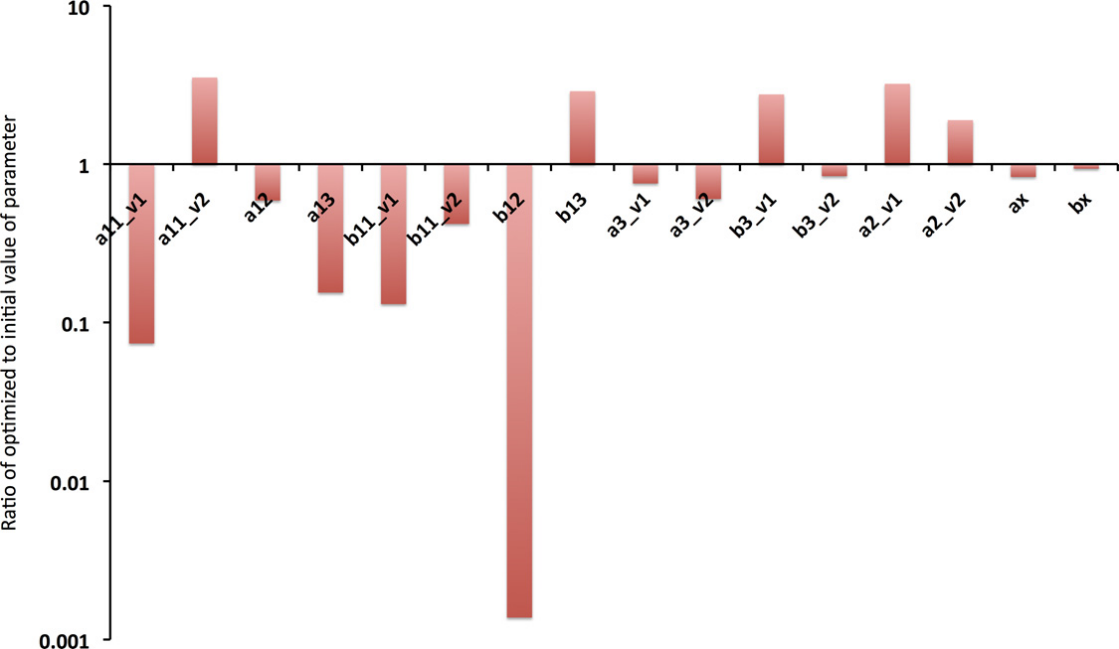
Ratio of optimized parameter to initial value. As can be seen, many parameters needed minimal optimization to accurately fit the wealth of data from multiple protocols. Some parameters, however, varied markedly from their initial value (e.g. b12). See **Table 1** and S1 Supplementary Information for the initial, and optimized parameter values.

The drug free Na^+^ channel model converged in 1235 iterations using convergence specifications of a tolerance of 0.01 for the change of the cost function, and 0.01 for the change in parameters. As can be seen in **Fig 3** (red traces), the optimized model effectively recapitulates the experimentally measured kinetics. It is also notable that some transition rates changed only a small amount during optimization. Nearly all of the optimized rate constants were within an order of magnitude of the initial value. Notably b12 changed by a factor of 1000; this likely suggests there is insufficient data to fully constrain this variable. **Fig 4** shows the ratio of the optimized parameter to the initial value of the parameter extracted from the literature.

These results suggest that while there is considerable variability between experimental datasets, optimization strategies can be used to formulate a model that broadly captures behaviors in multiple datasets, and can be used to probe model structure, effectively discarding unnecessarily complicated models (too many states) in favor of the simplest gating scheme (done here) that reasonably captures measured channel properties.

### Robustness of the drug-free model, sensitivity and parameter identifiably

There is a diverse literature base on parameter optimization, including analysis of uniqueness of fit, parameter identifiability etc. The reader is encouraged to review [22,23,25]. Briefly, a model is considered identifiable with regards to given experimental data, if it can reproduce that data using a unique set of parameters [25]. In practice, local (rather than global) identifiability is considered sufficient, as it is difficult or impossible to ensure global identifiability in all but the most simple of models. As noted by Fink [22], unidentifiable parameters make numerical optimization algorithms difficult, as optimized parameters become dependent on initial conditions.

We loosely followed the methods of Fink et al. by ensuring that our model was not over-parameterized and displayed no *a priori* unidentifiability (a condition whereby 1 parameter in the model can be recast as a combination of other parameters).

Because the model formulation represents a highly nonlinear system with multiple degrees of freedom and complex topology, we undertook a robustness testing strategy to determine if the algorithm returned to the ***same*** “global” minimum with increasingly large perturbations from the minimum starting point. We defined the results of the drug free optimization as our ***true*** “global” minimum; in other words, simulating the aforementioned protocols with those values would yield a cost function of zero.

**Fig 5:**
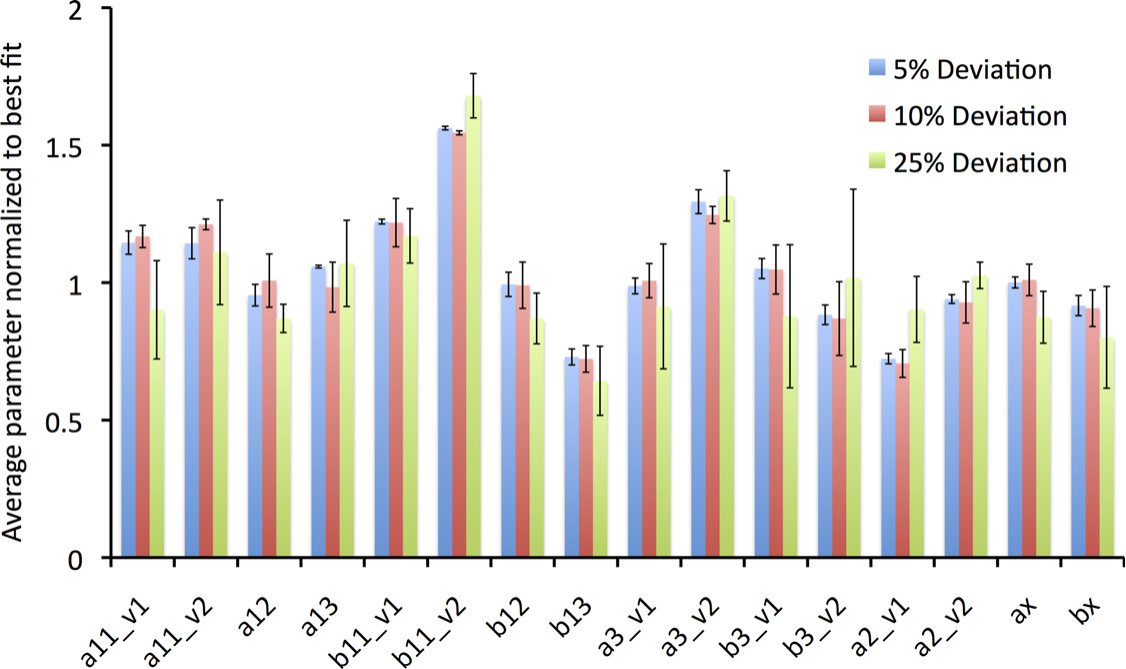
Robustness Analysis of Optimized Parameters. An optimization routine was set up such that a “true” global minimum was defined as the optimized parameters. The previously found optimized values were perturbed by a random number with a 5% (n = 3 runs), 10% (n = 3 runs), or 25% (n = 3 runs) deviation. The deviated parameters were used as the initial guesses, and the optimization algorithm was restarted. The graph shows the averaged parameter values from each set of runs, normalized to the optimized starting value. A value of 1 with no error bar would mean that the strategy found the exact optimized value. The error bars show ±1 standard deviation, normalized to the average value found from the runs. As can be seen, the algorithm performed best with smaller deviations from the optimum value, and many parameters were sufficiently constrained, even with large (25%) perturbations.

The parameter values were systematically perturbed away from their optimum value (again, defined as the optimized parameter values found as described above) by multiplying the parameter by a random number generated that was ± 5, 10, or 25% from the nominal value. This was done 3 times for each percentage perturbation. The optimization algorithm was restarted from the perturbed initial values and was assessed for how close it returned to the “optimum” (initial) values. **Fig 5** shows the ratio of the average normalized parameter after 3 runs to the “global minimum” parameter, and the standard deviation, normalized to the average. Stated differently, this robustness test is a measure of how well the algorithm performs when one knows the ***true*** global parameters, and how well the optimization strategy performs if the initial values are perturbed by 5, 10, or 25% away from the optimum value.

As can be seen in **Fig 5**, there are certain parameter values that are not well constrained by the experimental data. Thus, it is possible to derive multiple parameter sets that yield acceptable fits to the data, and of note, all runs of the optimization yielded parameter sets with an acceptably low cost function. It can also be seen, that the larger the perturbation away from the “global minimum”, the larger the discordance (variability) between the initial and ending parameter (e.g. compare magnitude of error bars between the 5% deviation and the 25% deviation). As new experimental data become available to further constrain the model, the optimization procedure should tend towards the true global minimum.

### Application of the procedure to drug blockade

One promising extension of state-dependent coupled ion channel models is for modeling the interactions of drugs with discrete conformational states of the ion channel [3,40]. We next describe the optimization process to tune parameters in an extended model of ion channel interactions with a drug. We briefly detail construction of a model of the cardiac Na^+^ channel interaction with the class I antiarrhythmic drug flecainide.

The optimization procedure follows from the drug free Na^+^ channel and makes use of measured pharmacokinetic properties of the drug to be simulated (e.g. diffusion rate, pKa for charge distribution etc., therapeutically relevant drug concentrations). A wide variety of experiments that capture features of drug blockade are used to constrain the model and include (1) slow, tonic block, (2) use-dependent block (frequency and concentration dependence), (3) steady-state availability in the presence of drug, and (4) recovery from drug bound states [3].

For example, the pKa of flecainide is 9.3 [10], yielding roughly 98% charged species at physiological pH. Diffusion of flecainide was measured to be ~5830 M^-1^ ms^-1^ [41], which we estimated as 5500 M^-1^ ms^-1^ in the computational model. The flecainide drug-channel model parameters for the on and off rates are derived from experiments, where possible, or computed from the available data. These include diffusion rates that indicate drug *on* rates “k_on_” = [drug]* D (diffusion rate) and affinities (Kd) to discrete conformations that determine drug *off* rates “k_off_” = Kd*D (diffusion rate).

**Fig 6:**
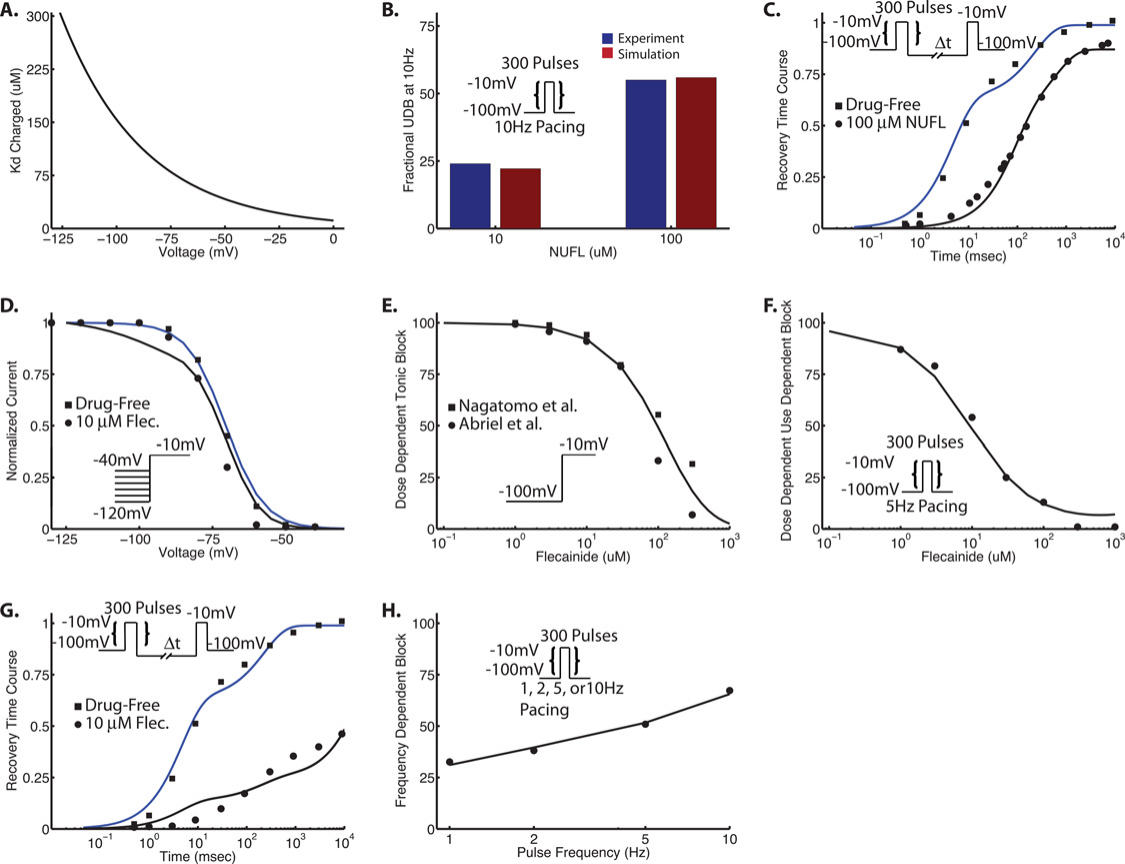
Flecainide drug binding. In panel (A,) a computed Kd curve is generated from Kd = Kd_0_*e^(-d*V*F/(R*T))^ with Kd_0 =_ 11.2 (Kd at 0 mV). Kd_-100mV_ = 175.8 μM. See **Table 3**. Panel B and C are the results from optimization of neutral rate constants for flecainide using a neutral flecainide derivative (NUFL). Panel (B) is use-dependent block at 10 Hz for 10 μM and 100 μM flecainide. Blue bars are experiment [10], and red bars are the result of the simulation. Panel C is recovery from UDB with 100 μM NUFL. Panels (D–H) are the results of the optimization for charged flecainide under a variety of protocols: (D)–steady state availability, (E)–tonic block (1-pulse block), (F)–use-dependent block (UDB), (G)–recovery from UDB, and (H)–frequency dependent use-dependent block. Protocols are shown as insets.

The model contains two binding schemes as described previously [3]: a charged, and a neutral flecainide drug binding scheme. While the full details are beyond the scope of this manuscript, the following is a brief summary on charge-state conformation specificity. The charged fraction of flecainide does not readily access inactivated states. Open state affinity for the charged form was derived from Kd values obtained from experiments measuring use-dependent blocking (UDB) affinity, an estimate of affinity to the open state [11]. Measured affinities (11.2 μM for flecainide) were defined as Kd_0_ –the Kd at 0 mV. Closed state affinity of charged drug was then calculated using Eyring rate theory for the voltage dependence of rate constants (Kd = Kd_0_*e^(-d*V*F/(R*T))^) [13]. For example, the Kd value at -100 mV for flecainide was computed to be 175.8 μM, which estimates charged drug affinity for the closed state (see **Fig 6A**).

The state specific affinities of neutral flecainide to closed and inactivated states were measured as 794 μM and 5.32 μM, respectively [10]. We used 800 μM and 5.4 μM in the computational model. Affinity of neutral flecainide to the open state was fit to UDB data in [10], and yielded a computed Kd of 400 μM.

The values noted above, and listed in **Table 3** were fixed and not allowed to change during the optimization procedure.

**Table 3:** Model State Specific Affinities of Drug to the Sodium Channel Flecainide.

	Charged	Neutral
Open state	Kd_0_ = 11.2 μM [11,42]	400 μM [10]
	Kd = Kd_0_*e^(-d*V*F/(R*T))^ [13]	
Closed states	Kd_-100mV_ = 175.8 μM (computed)	800 μM [10]
Inactivated states	N/A	5.4 μM [10]

d = fractional electrical charge = 0.7; F = Faraday’s constant = 96485.3415; R = Universal gas constant = 8314.472; T = Temperature (in K) = 295 for optimization; Kd_0_ = Kd at 0mV (measured affinity at 0 mV)

For the drug block model, the same optimization procedure as outlined above for drug-free channels was used. Five experimental protocols were used to constrain the model: steady state availability (SSA), tonic block (TB), use-dependent block (UDB), recovery from UDB (RUDB), and frequency-dependent UDB (FDUDB).

The computational model of flecainide contains 8 free charged drug rate constants (αx1, βx1, α13c, α22, β33, α33, α44, β44), and 8 free neutral drug rate constants (αx2, α13n, α_22, β_33, α_44, β_44, ki_on, ki_off); the rest of the drug rate constants are constrained by microscopic reversibility. Each rate constant is a scalar value of the drug free parameter. For example, αx1 = A*αx. The initial condition for the charged and neutral rates can be found in the **S1 Supplementary Information** and **S1 Fig**.

To simplify the problem, we split the optimization into two parts: charged, and neutral flecainide. To determine the neutral rate constants, we took advantage of a fully neutral analog of flecainide and first optimized the 8 neutral rates with UDB data at 10 Hz for 10 μM and 100 μM NUFL (**Fig 6B**), as well as recovery from UDB with 100 μM NUFL (**Fig 6C**). The initial values for the neutral rate constants were 1* drug free rate constant. After convergence, these rates were held constant while the charged rate constants were optimized over the five protocols listed above (SSA, TB, UDB, RUDB, FDUDB). For the charged fraction of flecainide, we started with our previously published model parameters [3]. As shown in **Fig 6D–6H**, the model achieved good fits to the experimental data. Further details on applications and predictions can be found in [3].

### Analysis of initial conditions

It is notable that the algorithm is not perfect, and has the potential to get stuck at local minima. Given the direct search nature of the algorithm (as compared to global optimization routines), it trades simplicity and speed for robustness. Starting from the converged neutral rates as noted above, we examined the dependence of the charged flecainide optimization on initial parameters. In addition to starting from our previously published results, we started the optimization from 3 additional starting vectors of initial guesses: a sequential strategy, selected inputs based on modeler intuition, and all 1’s (indicating that the drug bound rate = 1* drug free rate). As a reminder, drug binding rate is of the form: drug rate = parameter * drug free rate.

**Fig 7:**
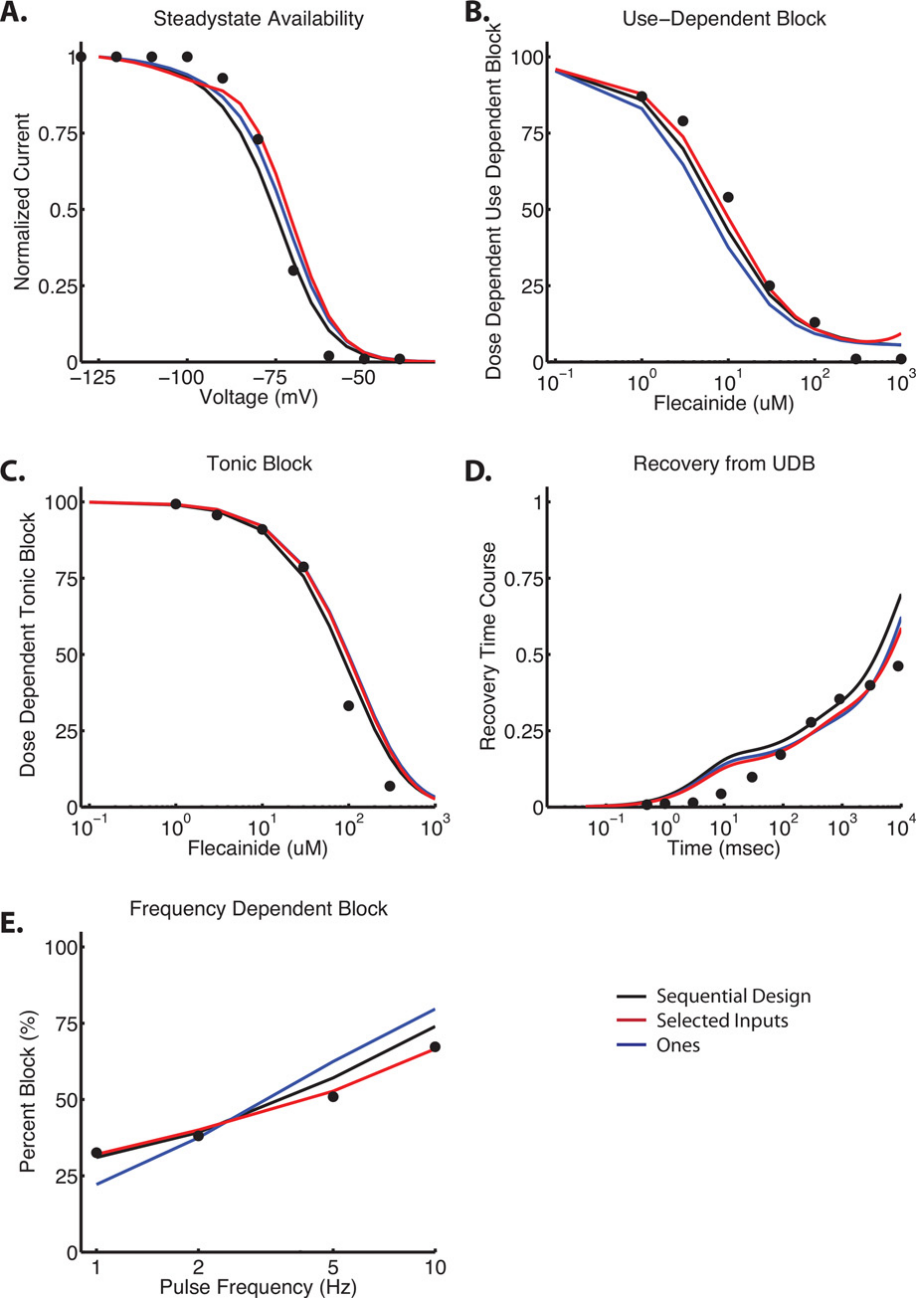
Analysis of initial conditions in charged flecainide model. Shown are the results of 3 additional optimization routines for charged flecainide, all starting from different initial conditions. The black trace is from a sequential design strategy of 400 initial iterations (100 each) of SSA, Block, RUDB, and FDUDB. The optimization was then continued to convergence. Red inputs indicated an empirically derived (“hand tuned”) set of initial guesses (see **Table 4**). Blue traces indicate an initial vector of all 1’s–indicating that the drug bound rate constant = 1* the drug free rate constant. See text for further details.

Our first trial implemented a sequential optimization in an attempt to “push” the system into a more reasonable parameter space, and to assess the dependence of sequential optimization on the final values. We started with four protocols–SSA, Block (including UDB and TB), frequency dependent block (FDUDB), and recovery (RUDB), and sequentially optimized each protocol for 100 iterations before adding in the second, third and fourth protocol. We implemented a full factorial design and, accordingly, ran 24 (4!) different iterations. For example, our first iteration started with SSA; after 100 iterations, RUDB was then added into the optimization routine (starting with the results after the 100 iterations of SSA). Iteration 101–200 optimized SSA and RUDB in parallel. Frequency dependent block was then added, and for iteration 201–300, SSA, RUDB, FDUDB were optimized in parallel. Finally, Block was included for iteration 301–400. This was repeated for each possible combination. The combination SSA, Block, RUDB, FDUDB gave the lowest value on the cost function, and thus we continued this parameter set to convergence (**Fig 7** –Black traces). This method gave acceptable fits to the data, but as compared to the best fit, the objective function was ~65% higher (e.g. total error on best fit was 165, vs. 272). See **Table 4**.

**Table 4:** Summary data of charged flecainide parameters starting from different initial conditions.

*Parameter*	*Best Fit Initial*	*Best Fit Converged*	*Ones Initial*	*Ones Converged*	*Sequential Initial*	*Sequential Converged*	*Selected Initial*	*Selected Converged*
**ax1**	5.7839E-05	1.0836E-05	1	2.4481E+00	1.9541E-02	1.5090E-02	1.0000E+00	1.4439E-01
**bx1**	1.6689E-08	4.2106E-08	1	1.7709E-02	4.3132E-04	1.2162E-09	1.0000E-04	3.6637E-04
**a13c**	3.6324E-03	2.4824E-03	1	3.6791E+00	1.6732E+02	5.6531E+02	1.0000E+00	3.4583E-03
**a22**	1.4847E+03	1.2663E+02	1	3.0744E-01	1.3239E-02	1.4486E-02	1.0000E+00	2.1129E+00
**b33**	1.7352E-06	4.8810E-06	1	5.2879E-01	2.7360E-02	2.3675E-02	1.0000E-04	4.8304E-08
**a33**	6.7505E-05	1.8309E-04	1	1.5913E+00	5.6535E-04	5.3763E-04	1.0000E-04	2.6205E-04
**a44**	2.4135E+00	2.5183E+00	1	4.6704E-09	7.7744E+00	9.9942E-02	1.0000E+00	2.3428E+00
**b44**	4.9001E-02	4.6378E-02	1	1.0091E+00	1.6783E+00	3.7766E+00	1.0000E+00	1.0219E-02
***Total Error***		1.6500E+02		4.8000E+02		2.7200E+02		1.7200E+02
***Iterartion***		432		256		311		388

Our second trial relied on “operator intuition” through analysis and interpretation of many data sets, and started with a vector of initial guesses that gave qualitatively good initial fits to the data, using order of magnitude estimates. Initial guesses are shown in **Table 4**, and the optimized model is shown in **Fig 7** –Red traces). As can be seen qualitatively, and quantitatively, this method gave nearly identical results our quantitatively “best” model (**Fig 6**). Notably, the total error was only 4% worse than our “best fit”.

Lastly, when we start the charged flecainide optimization from an initial vector of all 1’s (e.g. setting the drug bound rate equal to the drug free rate), the routine converges to a local minimum with unacceptably poor fits (**Fig 7** –Blue traces). The total error is almost 3x the magnitude of the “best fit” error.

## Discussion

Computer modeling and simulation of ion channels has become a widely used and largely accepted approach to study ion channel function. Models are increasingly extended to include the effects of perturbations such as the effects of mutations, regulation by intracellular signaling components and pharmacology. We recently developed a coupled state-dependent model of the cardiac Na^+^ channel to predict the interactions of antiarrhythmic drugs [3]. This model was the compilation of years of experimental research and development that suggested a multi-state kinetic mechanism of gating [2,13,19,26,39,43,44]. We then surveyed the literature to find voltage-clamp datasets that attempted to isolate specific molecular transitions and extracted rate constants for those transitions. Implementing a simple and relatively robust direct search numerical optimization strategy with no *a priori* weighting of protocols, we simultaneously fit multiple voltage-clamp datasets. The optimized model recapitulated many features of physiological gating, and was expanded for modeling drug-channel interactions.

Our strategy of parallel optimization of multiple datasets—so called ‘multi-objective fitting’ has been suggested to improve model outcome and credibility [24], parameter identifiability [24,25], and specifically guards against *a priori* weighting of kinetic transitions. The method proposed by Fink et al. [22,45] to create one single protocol for channel gating that is sufficient to capture the most essential gating kinetics is of course ideal. However, in practice, the modeler finds herself with multiple distinct datasets from different laboratories. Multi-objective fitting can be used to “meld together” a wide range of data from the experimental literature into a cohesive computational framework that summarizes decades of experiment and understanding [18]. Lastly, by optimizing multiple datasets in parallel (e.g. activation, inactivation, recovery), no specific transition is favored in the optimization process. While Menon et al. derive an ingenious state mutating genetic algorithm to optimize *both* topology and kinetic parameters, their sequential goal-programming technique defines an implicit hierarchy to certain kinetic transitions [21].

With regards to local and global identifiability, our implementation of the Nelder Mead method likely finds a local minimum, and it is virtually impossible to prove a global minimum [23,25]. It has been shown that multiple optimization strategies, and even multiple iterations of the *same* optimization strategy do not yield exactly the same results, indicating a non-unique parameter set (especially if the model is over-parameterized). However, we [3], and others [24], have previously shown that multiple parameter sets that accurately fit the data do not necessarily affect the robust output of the model, but then parameter sets and model behaviors need to be subject to sensitivity analysis. As noted by Hui et al. [25], identifiability must be balanced against convergence rate and total number of model evaluations to find the least complex model that is sufficiently predictive for a given application.

Additional data designed to measure specific kinetic transitions in the model will likely be useful to increasingly constrain the model parameters to a truly unique solution, but *only if* the experiment is *truly* isolating a specific kinetic transition. Our analysis suggests that pure isolation of specific kinetic processes by electrophysiological techniques for channels is far from perfect [22,46]. For example, recovery from inactivation time course likely represents additional processes–channels opening for the first time, inactivating and recovering, channels deactivating and then reactivating again etc. Although the time-course may be dominated by one kinetic transition, the full time course may more likely be a mix of multiple processes [11]. One possibility is that new techniques that enable more sensitive measurements of individual transitions of proteins–especially those that don’t produce distinct ionic currents (i.e. deactivation, activation, recovery)–will allow for better constraints of individual rate constants.

An additional point that warrants discussion is the robustness of the model to predict emergent pharmacology given uncertainty of initial parameters. In addition to the analysis described above, one strategy that we [3], and others have utilized is a sensitivity analysis of the model input parameters to a well-defined model output, namely ionic current. We have shown previously that the model is not overly sensitive to nominal inputs. One area of future research is extending a sensitivity analysis of both model input (parameters) and output at each space and time scale (e.g. ionic current, cellular membrane potential, and tissue dynamics). For example, Yang [47] simulated a population of single cells by varying all model parameters randomly by 20% of their nominal values and ascertained action potential duration at 90% repolarization (APD_90_) as the model test output for the simulated population. This simulation reflects an efficient way to observe responses in a population of action potentials with varying parameters. Lastly, Yang et al. [47] induced beat-to-beat APD variability by adding noise currents to the total membrane potential throughout the simulated time course, and was able to determine the sensitivity of the model output to an applied perturbation. Thus, predictive models should undergo a thorough sensitivity analysis at each stage of development to ensure that the predictions of the model are valid and applicable.

Finally, our results are consistent with the notion that final parameters exhibit an important dependence on initial guesses; this likely results from either an incompletely constrained model, an over-determined system, or insufficient data to constrain the model. The closer the initial guesses are to the “correct” parameters, the more robust the optimization. In the absence of reasonably “good” initial guesses, the investigator might consider utilizing multiple randomly generated initial parameters to allow statistical tests of goodness of fit to the data. The initial condition sets that result in a predetermined user defined cost-function cutoff can be subjected to additional optimization tests, examined for sensitivity, and used in simulations that can be subject to validation tests with additional data sets in order to choose the final parameter set.

### Limitations

The approach we describe in the paper is not without limitations; while the Nelder Mead method is relatively easy to implement, it suffers from being relatively “slow”, requiring many iterations to converge (in the drug free Na^+^ channel described here, over 1250 iterations required upwards of 30 hours of computational time on an Intel Xeon 3.0 GHZ 8-core Mac Pro desktop computer). Because it is a direct search algorithm, there is potential for the routine to get stuck at a local minimum far from an acceptably optimized parameter set. We have identified a few strategies to help overcome this pitfall. One can restart the simulation after it hits a local minimum by perturbing the parameters at that point in the optimization (e.g. a random perturbation of each parameter by ± 10%), and then restarting the simulation. This can serve to “kick-start” the simulation and potentially overcome the local minimum. In addition, the appropriate selection of experimental protocols that focus on as few molecular transitions as possible, yielding initial guesses of parameters that are relatively close to the optimized value help to achieve an acceptable parameter set. It is also possible to use a “hybrid” strategy using empirically derived initial values that start the optimization qualitatively closer to an acceptable fit, and then use parameter optimization to achieve a quantitatively best fit. This strategy can help the modeler to derive a robust model that recapitulates the known experimental data.

There are other numerical strategies to avoid local minima–namely utilizing more robust “global” optimization methods such as genetic algorithm, multi-start algorithms, and simulated annealing, for example. While these methods are fairly easy to implement for the expert user (especially utilizing the built-in functionality within the Global Optimization™ and Parallel Computing™ toolboxes), they may not be accessible to non-experts and require tremendous increases in computational power. For example, the genetic algorithm implementation in MATLAB starts with a default generation value of 100*number of parameters, and 200 for an initial population size. With our model, this would require cloud or cluster computing functionality.

It is also important to note that modeling is by definition, a simplified representation of the underlying physiology. Connection to the protein structure is not an emergent property of the modeling but dependent on the modeler’s definition of the model topology [22]. There is also the possibility that model structure does not reflect molecular reality (e.g. the Na^+^ channel model by Menon requires that recovery into a closed state necessitating a transition through an open state [21]). Thus, careful application of the model with knowledge of the experimental biophysical data, and the conclusions drawn from model predictions must be realized with these limitations.

Lastly, the results of this study suggest that optimization is just one tool in the arsenal of simulation of drug function. To date, there is no “silver-bullet”, nor single best approach to deriving an ion channel model. Each model, and application thereof, requires careful analysis and consideration of the potential limitations implicit within its construction. The model needs to be as complex as is required for the problem of interest, but ideally, not more complex, nor simpler.

### Conclusions

Here, we present a detailed approach to a numerical optimization strategy to derive functional models of ion channels from experimental function data, and extend the framework to focus on a clinical application of pharmacokinetic modeling–ion channel drug interaction. We have previously used a comparable model in a multiscale framework to predict antiarrhythmic drug action and safety [3,40]. Since efficient methods for preclinical proarrhythmia assessment of candidate compounds affecting cardiac ion channels are currently lacking, modeling and simulation approaches that extend the types of model frameworks described here may comprise a plausible first screening step [48]. The approach has also been used to understand common mechanisms of inherited and acquired arrhythmia, with exploitation of common pathways for therapeutic drug targeting [40]. While the state-dependent ion channel model framework has focused on cardiac ion channel blockade, the methodology is potentially applicable in other diseases of excitability, disturbed ion channel function, and predictive pharmacology such as in epilepsy and pain.

The advent of novel, high-throughput electrophysiology [49], when combined with *in-silico* predictive models also has the potential to dramatically shorten the drug development cycle by providing early predictions for which agents merit further testing in higher dimensions and with experiments. It is hoped that a hybrid strategy can be scaled for high throughput screening and high sensitivity, which will be able to dramatically reduce costs by discarding compounds with unforeseen side effects much earlier in development.

Finally, as our understanding of cardiac physiology and the drug channel interaction becomes further refined, it may be possible to use this model framework to “reverse engineer” an “ideal” compound with specific biophysical properties that will be predicted to behave and produce specific antiarrhythmic effects. *In-silico* combinatorial approaches can then be used to guide synthesis of those compounds to validate model predictions.

## Supporting Information

S1 Fig Model schematic of the drug-channel interaction. Functional interactions of a local anesthetic with the cardiac Na channel are complex and determined by drug charge, channel conformation specific binding, gating kinetics of drug bound channel, and time and voltage dependence of recovery from drug block. Panel (**A**) shows a schematic indicating model assumptions of drug accessibility. Local anesthetics have two forms at physiological pH: neutral and protonated. Neutral drug diffuses through the membrane and may migrate into the receptor via a hydrophobic pathway. Neutral drug binds to all conformational states, with low affinity to closed and open states and markedly higher affinity for inactivated states (see State Specific Affinities in **Table 2**). Charged drugs obtain binding site access from inside only (hydrophilic pathway). Charged drug exhibits high voltage dependent affinity to the open state, low affinity to closed states and does not readily access inactivated states (see **Table 2**). Panel (**B**) is the Markovian model representation of the drug Na channel interaction. The drug free channel has 8 distinct states (top two rows of states shown in black), any of which can exist as a drug-bound conformation. There are two modes of drug bound channel states–red lines indicate entry or egress from a mode of charged drug bound states denoted by a red D+. Green lines indicate entry or egress from neutral drug-bound states denoted by a green D. Gating transitions that occur subsequent to drug binding may be affected by presence of drug. The flecainide model includes an additional inactivated, trapped state (DIT) [50]. Transition arrows were omitted from IC3→DIC3, IC2→DIC2, IF→DIF for clarity (blue box). Figure reproduced from [3].
(EPS)

S1 Supplementary Information This file contains rate constant derivations, vectors of initial guesses and optimized parameters, as well as brief explanation of the optimization code found within S1 Optimization Code. (DOCX)

S1 Optimization Code This .zip file contains all of the optimization code used in the article. (ZIP)
