# Supplementary material for: Parameterization for In-Silico Modeling of Ion Channel Interactions with Drugs
Source: PLoS One. 2016 Mar 10;11(3):e0150761. doi: 10.1371/journal.pone.0150761 (PMC4786197; doi:10.1371/journal.pone.0150761)
Supplement: S1 Supplementary Information — (DOCX) [file pone.0150761.s002.docx]

**Parameterization for *in-silico* modeling of ion channel interactions with drugs**

Jonathan D. Moreno, M.D., Ph.D., Timothy J. Lewis, Ph.D., and Colleen E. Clancy, Ph.D.

**SUPPLEMENTARY INFO:**

**Rate constant derivations:**

Below are the original data, curve fits and vector of initial guesses for the WT Na^+^ channel model as described in the manuscript.

***Recovery from inactivation (α3)***

| ***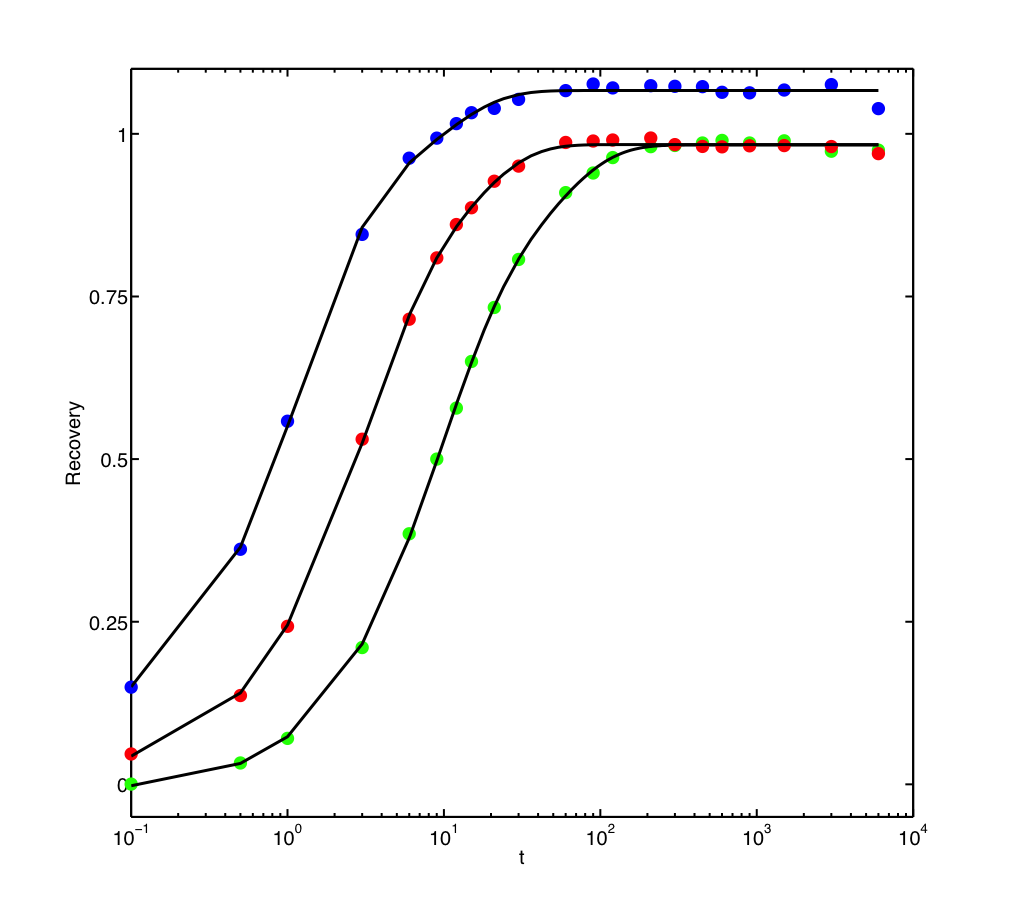***  ***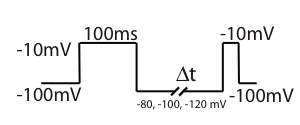***  Shown above, are recovery curves at -120mV (blue), -100mV (red), and -80mV (green) for the WT Na channel. A 100ms test pulse was elicited to -10mv for 100ms, the channel was allowed to recover at the given test voltage (-80, -100, -120mV) for the indicated time, and then was repulsed to -10mV. Plotted is the ratio of the 2^nd^ test pulse to the first (a standard 2 pulse protocol as shown above). Below, are the parameters for the curves of best fit to the equation: Recovery(t) = Y – A1*exp(-t/τ1) – A2*exp(-t/τ2): | | |
| --- | --- | --- |
| **-80 mV (Green dots)**  General model:  f(t) = Y-A1*exp(-t/tau1)-A2*exp(-t/tau2)  Coefficients (with 95% confidence bounds):  A1 = 0.6758 (0.6203, 0.7312)  A2 = 0.3182 (0.2617, 0.3746)  Y = 0.9829 (0.9789, 0.9869)  tau1 = 8.29 (7.537, 9.044)  tau2 = 42.62 (35.41, 49.84)  Goodness of fit:  SSE: 0.000437  R-square: 0.9998  Adjusted R-square: 0.9998  RMSE: 0.005226 | **-100 mV (Red dots)**  General model:  f(t) = Y-A1*exp(-t/tau1)-A2*exp(-t/tau2)  Coefficients (with 95% confidence bounds):  A1 = 0.3117 (0.2248, 0.3986)  A2 = 0.6548 (0.571, 0.7387)  Y = 0.9834 (0.9795, 0.9874)  tau1 = 12.47 (9.534, 15.41)  tau2 = 2.681 (2.305, 3.057)  Goodness of fit:  SSE: 0.0005914  R-square: 0.9997  Adjusted R-square: 0.9996  RMSE: 0.00608 | **-120 mV (Blue dots)**  General model:  f(t) = Y-A1*exp(-t/tau1)-A2*exp(-t/tau2)  Coefficients (with 95% confidence bounds):  A1 = 0.764 (0.6933, 0.8347)  A2 = 0.2186 (0.1452, 0.292)  Y = 1.067 (1.06, 1.073)  tau1 = 1.158 (0.985, 1.33)  tau2 = 8.417 (5.49, 11.34)  Goodness of fit:  SSE: 0.001531  R-square: 0.9989  Adjusted R-square: 0.9986  RMSE: 0.009783 |

Using the methods of Colquhoun and Hawkes [1], one can derive k1, k2, L1, L2 as follows:

| -120mV: A1 = 0.76, A2 = 0.22, L1 = 1/τ1 = (1/1.16), L2 = 1/τ2 = (1/8.4)  -100mV: A1 = 0.65, A2 = 0.31, L1 = 1/τ1 = (1/2.68), L2 = 1/τ2 (1/12.47)  -80mV: A1 = 0.68, A2 = 0.32, L1 = 1/τ1 = (1/8.3), L2 = 1/τ2 = (1/42.63)  k2 = A1 + A2  k1 = (L1*L2)/k2  Given this formulation, α3 is proportional to k1; a plot of k1 vs V is below; the line of best fit is set to α3(V). | |
| --- | --- |
| **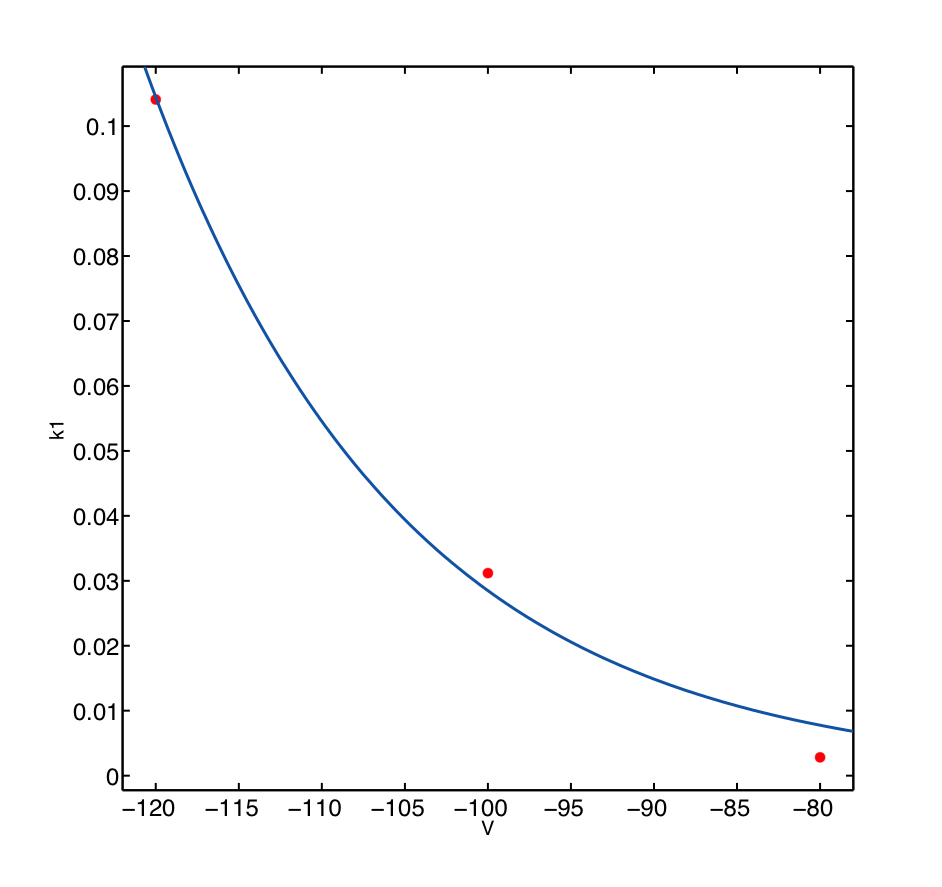** | |
| ***V*** = [-120; -100; -80]  ***k1*** = [0.1041; 0.031169; 0.0028263];  General model Exp1:  f(x) = a*exp(b*x)  Coefficients (with 95% confidence bounds):  a = 4.291e-05 (-0.0005392, 0.000625)  b = -0.06498 (-0.1794, 0.04944) | Goodness of fit:  SSE: 3.175e-05  R-square: 0.9942  Adjusted R-square: 0.9884  RMSE: 0.005635  α3 = 4.291E-05*exp(-V/15.39)  **A = 4.291E-05, B = 15.39** |

***Closed state inactivation (β3)***

The original data were extracted from Goldman et al. [2]

| 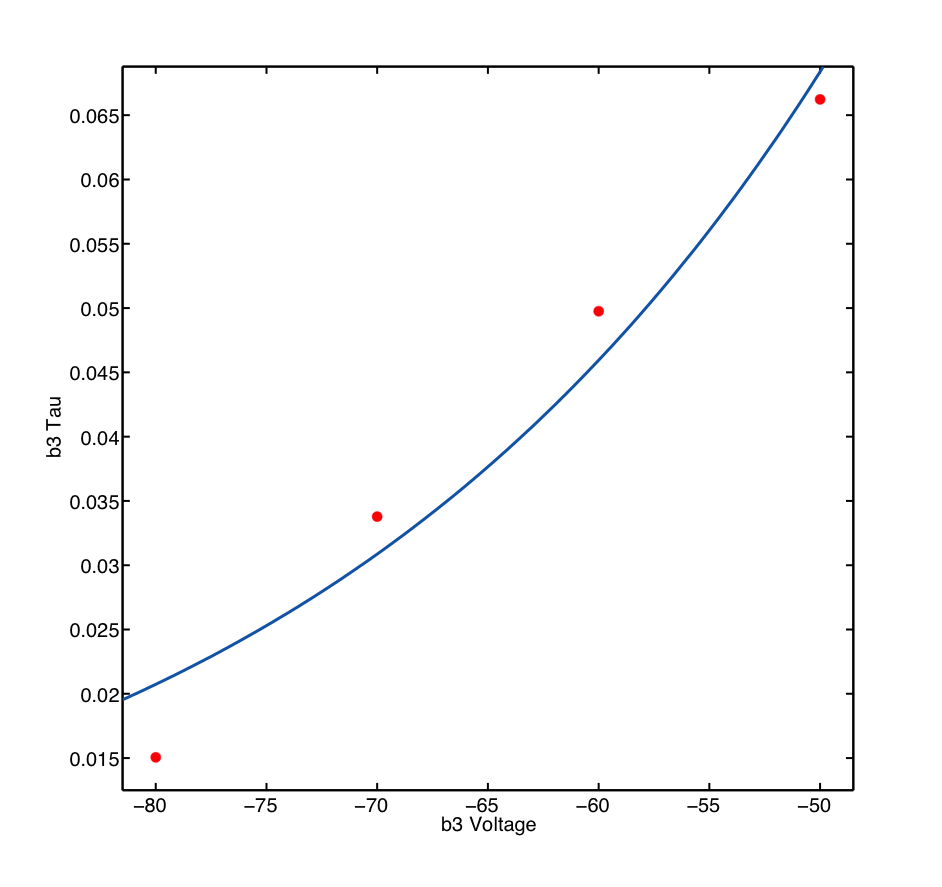 | |
| --- | --- |
| ***b3_Voltage*** = [-80; -70; -60; -50]';  ***b3_Tau_inverse*** = [1/66.4; 1/29.6; 1/20.1; 1/15.1]';  General model Exp1:  f(x) = a*exp(b*x)  Coefficients (with 95% confidence bounds):  a = 0.4996 (-0.3448, 1.344)  b = 0.03977 (0.01021, 0.06933) | Goodness of fit:  SSE: 5.99e-05  R-square: 0.9583  Adjusted R-square: 0.9375  RMSE: 0.005473  β3 = 0.4996*exp(V/25.14)  **A = 0.4996, B = 25.14** |

***Fast inactivation (α2)***

Inactivation from the open state (**α2** corresponding to O 🡪IF) was from Yue and Marban [3].

| 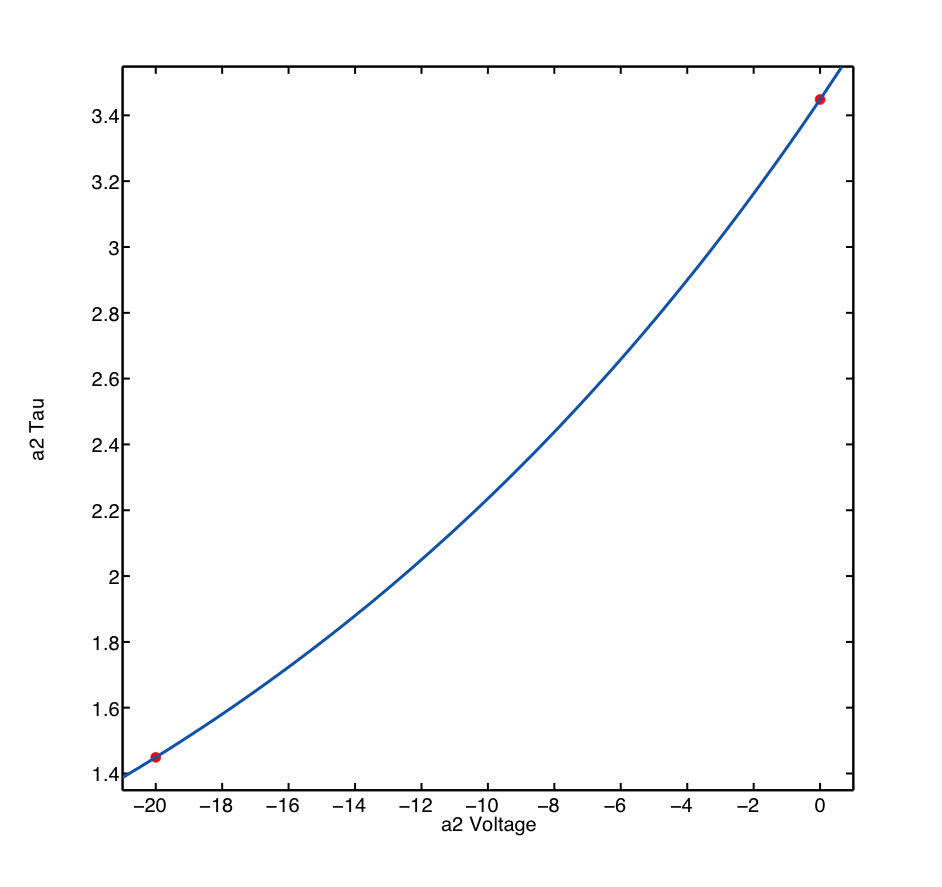 | |
| --- | --- |
| ***a2_Voltage*** = [-20, 0]';  ***a2_tau_inverse*** = [1/0.69; 1/0.29]';  General model Exp1:  f(x) = a*exp(b*x)  Coefficients:  a = 3.448  b = 0.04334 | Goodness of fit:  SSE: 1.972e-31  R-square: 1  Adjusted R-square: NaN  RMSE: NaN  a2 = 3.448*exp(V/23.07)  **A = 3.448, B = 23.07** |

***Recovery from fast inactivation (β2)***

As noted in the paper, this rate constant was constrained by microscopic reversibility [4]:


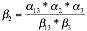


***Slow inactivation (entry – αx; exit βx)***

As mentioned in the paper, Slow inactivation from the open state (αx corresponding to O→IS) was from Lawrence et al. [5] which measured slow inactivation at -20 mV = 0.111/ms, corresponding to ≈ α2/20. Recovery from slow inactivation (βx corresponding to IS→O) was initially set at a3/45.

***Vector of initial guesses and optimized values for WT Drug-Free Model:***

| **Parameter** | **Initial** | **Final** |
| --- | --- | --- |
| **a11_v1** | 0.1027 | 7.6178e-03 |
| **a11_v2** | 9.3 | 3.2764e+01 |
| **a12** | 1 | 5.8871e-01 |
| **a13** | 1 | 1.5422e-01 |
| **b11_v1** | 19.832 | 2.5898e+00 |
| **b11_v2** | 20.3 | 8.5072e+00 |
| **b12** | 1.0 | 1.3760e-03 |
| **b13** | 1.0 | 2.8880e+00 |
| **a3_v1** | 4.301E-05 | 3.2459e-05 |
| **a3_v2** | 15.934 | 9.5951e+00 |
| **b3_v1** | 0.4996 | 1.3771e+00 |
| **b3_v2** | 25.14 | 2.1126e+01 |
| **a2_v1** | 3.448 | 1.1086e+01 |
| **a2_v2** | 23.07 | 4.3725e+01 |
| **ax** | 0.05 | 4.1476e-02 |
| **bx** | 0.0222 | 2.0802e-02 |

**Flecainide Drug Binding**

***Vector of initial guesses and optimized values for the flecainide drug binding model***

|  | **Form** | **Initial** | **Final** |
| --- | --- | --- | --- |
| **ax1** | A*ax | 5.7839e-05 | 1.0836e-05 |
| **bx1** | A*bx | 1.6689e-08 | 4.2106e-08 |
| **a13c** | A*a13 | 3.6324e-03 | 2.4824e-03 |
| **a22** | A*a2 | 1.4847e+03 | 1.2663e+02 |
| **b33** | A*b3 | 1.7352e-06 | 4.8810e-06 |
| **a33** | A*a3 | 6.7505e-05 | 1.8309e-04 |
| **a44** | A*a4 | 2.4135e+00 | 2.5183e+00 |
| **b44** | A*b4 | 4.9001e-02 | 4.6378e-02 |
|  |  |  |  |
| **ax2** | A*ax | 1 | 1.6936e+01 |
| **a13n** | A*a13 | 1 | 1.0714e-01 |
| **a_22** | A*a2 | 1 | 5.9858e-01 |
| **b_33** | A*b3 | 1 | 5.6001e-01 |
| **a_44** | A*a4 | 1 | 2.8919e-07 |
| **b_44** | A*b4 | 1 | 1.2546e+01 |
| **ki_on** | A*k_on | 1 | 6.6880e-01 |
| **ki_off** | A*k_on | 1 | 5.2802e-06 |

*****Note, the initial rates above are from Moreno et al.[6].

**Brief explanation of the optimization code**

The following is a brief summary of the files contained within the code, when to invoke them, and what they are used for.

***Global_test.m***

- This is the main script file, where the initial conditions for the entire optimization are stored. From this header, a pool of MATLAB workers is initiated, the files are compiled (using the *mex* command), and the initial conditions, and bounds are set.
- The program is run from this header file

***WT_REDUCED_CHANNEL_Sim_Exp_Norm.m***

- This file contains the objective function for the drug-free WT channel model, and is where the parallelization takes place. From this file, each protocol is sent to a MATLAB worker, and the result of the simulation is compared to the experiment
- “Total_Error” at the bottom of the script is the objective function to be minimized

***WT_Drug_Sim_Exp_FLEC.m***

- This file contains the objective function for the drug-channel interaction, is where the parallelization takes place, and where the objective function is defined (similar to WT_REDUCED_CHANNEL_Sim_Exp_Norm.m above)

***Global_test_SEQ.m***

- This file is the main header file, when sequential optimization is required.
- From this script, a pool of MATLAB workers is initiated, the files are compiled (using the *mex* command), and the initial conditions and bounds are set.

***WT_Drug_Sim_Exp_FLEC_SEQ.m***

- This file is similar to WT_Drug_Sim_Exp_FLEC.m, except that the optimization is run sequentially, first with 1 protocol, then 2 protocols etc.

***fminsearchbnd.m***

- This is the bounded Nelder Mead algorithm
- Downloaded from http://www.mathworks.com/matlabcentral/fileexchange/8277-fminsearchbnd--fminsearchcon

***Global_Variables.h***

- This file contains the global variables used in the C++ code, where the protocols are simulated

**WT_Flec_implicit.h**

- This file is the WT drug channel model, where the differential equations describing the kinetic transitions of the different states of the Na channel model are described
- The input parameters to be optimized are further defined here

***main_SSA.cpp; main_ACT.cpp; main_RFI.cpp; main_RUDB.cpp; main_TAU.cpp***

- The above protocols are used in the WT drug-free fitting
- SSA – steady state availability, ACT – steady state activation, RFI – recovery from inactivation, RUDB – recovery from use-dependent block, tau – time constant (τ) to inactivation

***main_BLOCK.cpp; main_FDUDB1.cpp; main_FDUDB2.cpp; main_FDUDB_NUFL.cpp;***

- The above protocols are used in the drug-binding fitting
- Note, that FDUDB is split into two protocols, so that it can be sent to two MATLAB workers to speed up the simulation (1Hz for FDUDB1; 2, 5, and 10Hz are simulated with FDUDB2)
- Block – concentration dependent tonic, and use-dependent block, FDUDB – frequency dependent block, cell – single cell simulation

**REFERENCES**

1. Colquhoun, D. and A.G. Hawkes, *On the stochastic properties of single ion channels.* Proc R Soc Lond B Biol Sci, 1981. **211**(1183): p. 205-35.

2. Goldman, L., *Sodium channel inactivation from closed states: evidence for an intrinsic voltage dependency.* Biophys J, 1995. **69**(6): p. 2369-77.

3. Yue, D.T., J.H. Lawrence, and E. Marban, *Two molecular transitions influence cardiac sodium channel gating.* Science, 1989. **244**(4902): p. 349-52.

4. Colquhoun, D., et al., *How to impose microscopic reversibility in complex reaction mechanisms.* Biophys J, 2004. **86**(6): p. 3510-8.

5. Lawrence, J.H., et al., *Sodium channel inactivation from resting states in guinea-pig ventricular myocytes.* J Physiol, 1991. **443**: p. 629-50.

6. Moreno, J.D., et al., *A computational model to predict the effects of class I anti-arrhythmic drugs on ventricular rhythms.* Sci Transl Med, 2011. **3**(98): p. 98ra83.
