# Supplementary material for: Parameterization for In-Silico Modeling of Ion Channel Interactions with Drugs
Source: PLoS One. 2016 Mar 10;11(3):e0150761. doi: 10.1371/journal.pone.0150761 (PMC4786197; doi:10.1371/journal.pone.0150761)
Supplement: S1 Optimization Code — (ZIP) [file pone.0150761.s003.zip › OPT Code/Code_notes.docx]

The following is a brief summary of the files contained within the code; when to invoke them, and what they are used for.

***Global_test.m***

- This is the main header file, where the initial conditions for the entire optimization are stored. From this header, a pool of MATLAB workers is initiated, the files are compiled (using the *mex* command), and the initial conditions, and bounds are set.
- The program is run from this header file

***WT_REDUCED_CHANNEL_Sim_Exp_Norm.m***

- This file contains the objective function for the drug-free WT channel model, and is where the parallelization takes place. Each protocol is sent to a MATLAB worker, and the result of the simulation is compared to the experiment
- “Total_Error” at the bottom of the script is the objective function to be minimized

***WT_Drug_Sim_Exp_FLEC.m***

- This file contains the objective function for the drug-channel interaction, is where the parallelization takes place, and where the objective function is defined (similar to WT_REDUCED_CHANNEL_Sim_Exp_Norm.m above)

***Global_test_SEQ.m***

- This file is the main header file, when sequential optimization is required.
- From this header, a pool of MATLAB workers is initiated, the files are compiled (using the *mex* command), and the initial conditions, and bounds are set.

***WT_Drug_Sim_Exp_FLEC_SEQ.m***

- This file is similar to WT_Drug_Sim_Exp_FLEC.m, except that the optimization is run sequentially, first with 1 protocol, then 2 protocols etc.

***fminsearchbnd.m***

- This is the bounded Nelder Mead algorithm

***Global_Variables.h***

- This file contains the global variables used in the C++ code, where the protocols are simulated

**WT_Flec_implicit.h**

- This file is the WT drug channel model, where the differential equations describing the kinetic transitions of the different states of the Na channel model are described
- The input parameters to be optimized are further defined here

***main_SSA.cpp; main_ACT.cpp; main_RFI.cpp; main_RUDB.cpp; main_TAU.cpp***

- The above protocols are used in the WT drug-free fitting

***main_BLOCK.cpp; main_FDUDB1.cpp; main_FDUDB2.cpp; main_CELL.cpp***

- The above protocols are used in the drug-binding fitting
- Note, that FDUDB is split into two protocols, so that it can be sent to two MATLAB workers to speed up the simulation (1Hz for FDUDB1; 2, 5, and 10Hz are simulated with FDUDB2)
